# Change in Brain Magnetic Resonance Spectroscopy after Treatment during Acute HIV Infection

**DOI:** 10.1371/journal.pone.0049272

**Published:** 2012-11-16

**Authors:** Napapon Sailasuta, William Ross, Jintanat Ananworanich, Thep Chalermchai, Victor DeGruttola, Sukalaya Lerdlum, Mantana Pothisri, Edgar Busovaca, Silvia Ratto-Kim, Linda Jagodzinski, Serena Spudich, Nelson Michael, Jerome H. Kim, Victor Valcour, for the RV254/SEARCH 010 protocol teams

**Affiliations:** 1 Huntington Medical Research Institutes, Pasadena, California, United States of America; 2 Southeast Asia Research Collaboration with Hawaii (SEARCH) - Thailand, Bangkok, Thailand; 3 Faculty of Medicine, Chulalongkorn University, Bangkok, Thailand; 4 The HIV Netherlands Australia Thailand Research Collaboration (HIV-NAT), The Thai Red Cross AIDS Research Center, Bangkok, Thailand; 5 Military HIV Research Program, Walter Reed Army Institute of Research, Silver Spring, Maryland, United States of America; 6 Department of Biostatistics, Harvard School of Public Health, Boston, Massachusetts, United States of America; 7 Department of Neurology, Memory and Aging Center, University of California San Francisco, San Francisco, California, United States of America; 8 Department of Neurology, Yale School of Medicine, New Haven, Connecticut, United States of America; 9 Division of Geriatric Medicine, Department of Medicine, University of California San Francisco, San Francisco, California, United States of America; South Texas Veterans Health Care System and University Health Science Center San Antonio, United States of America

## Abstract

**Objective:**

Single voxel proton magnetic resonance spectroscopy (MRS) can be used to monitor changes in brain inflammation and neuronal integrity associated with HIV infection and its treatments. We used MRS to measure brain changes during the first weeks following HIV infection and in response to antiretroviral therapy (ART).

**Methods:**

Brain metabolite levels of N-acetyl aspartate (NAA), choline (tCHO), creatine (CR), myoinositol (MI), and glutamate and glutamine (GLX) were measured in acute HIV subjects (n = 31) and compared to chronic HIV+individuals (n = 26) and HIV negative control subjects (n = 10) from Bangkok, Thailand. Metabolites were measured in frontal gray matter (FGM), frontal white matter (FWM), occipital gray matter (OGM), and basal ganglia (BG). Repeat measures were obtained in 17 acute subjects 1, 3 and 6 months following initiation of ART.

**Results:**

After adjustment for age we identified elevated BG tCHO/CR in acute HIV cases at baseline (median 14 days after HIV infection) compared to control (*p* = 0.0014), as well as chronic subjects (*p* = 0.0023). A similar tCHO/CR elevation was noted in OGM; no other metabolite abnormalities were seen between acute and control subjects. Mixed longitudinal models revealed resolution of BG tCHO/CR elevation after ART (p = 0.022) with tCHO/CR similar to control subjects at 6 months.

**Interpretation:**

We detected cellular inflammation in the absence of measurable neuronal injury within the first month of HIV infection, and normalization of this inflammation following acutely administered ART. Our findings suggest that early ART may be neuroprotective in HIV infection by mitigating processes leading to CNS injury.

## Introduction

Infection with human immunodeficiency virus-1 (HIV) remains a major health issue affecting 34 million people worldwide, with 2.7 million new infections reported in 2010 [1]. HIV enters the central nervous system (CNS) within days of initial exposure, based on limited reports in humans and studies from animal models employing simian immunodeficiency virus (SIV) [2], [3]. Lacking the CD4+cell surface receptor, neurons are not appreciably infected by HIV. Rather, an inflammatory response ensues, involving microglial cells and perivascular macrophages and leading to neuronal dysfunction and, ultimately, neuronal loss [4]. Limited human studies completed within the first year of infection (“primary” rather than “acute” infection) suggest early CNS involvement [5], [6]. The extent and timing of CNS invasion is not precisely known in humans, but is increasingly important as the field focuses on HIV eradication strategies that require a clear understanding of when viral reservoirs are established and how early intervention may impact these reservoirs. Strategies to address chronic complications of HIV may also be informed by understanding the timing and characteristics of early CNS involvement.

Infection and inflammation of the CNS is linked to neurological and cognitive findings in chronic HIV. Cerebrospinal fluid (CSF) HIV RNA levels are more valuable in predicting neuropsychological testing impairment 6 months following initiation of ART than are plasma HIV RNA levels, CD4+lymphocyte counts, or Centers for Disease Control and Prevention disease stage classification [7]. Despite suppressive ART, many patients experience mild deficits of cognitive and motor ability that impact function in later stages of infection [8]. The extent to which events of acute HIV influence long-term consequences in the CNS is incompletely understood.

Non-invasive ^1^H-proton magnetic resonance spectroscopy (MRS) measures signals from hydrogen atoms specific to the molecular structure of the metabolite in which they are contained. Brain MRS studies can document the extent of inflammation underlying HIV, disease progression and treatment response. Neurons can be distinguished by the relative level of N-acetyl-aspartate (NAA), because it is synthesized within the brain almost exclusively by neurons from aspartate and acetyl coenzyme A [9]. Decreased NAA is noted in association with HIV-associated dementia (HAD), cognitive impairment, neurological symptoms and among medically asymptomatic HIV+individuals [10], [11], [12]. Increased myoinositol (MI) is observed in HIV dementia and thought to represent gliosis, whereas increased total choline (tCHO) in this setting is thought to represent infiltration of inflammatory cells [13], [14]. Glutamate (GLU), a major brain excitatory neurotransmitter, is associated with HIV-induced neurotoxicity where excessive activation of N-methyl-D-aspartate receptors results in increased extracellular glutamate and neuronal cell death [15], [16]. At 1.5 tesla magnet strength, glutamate cannot be confidently partitioned from glutamine (GLN) and is combined for analyses (GLX).

The primary goal for this study was to examine brain MRS metabolites during the first month of HIV infection. We sought to define the extent of brain parenchymal inflammation and neuronal injury and to examine changes in these metabolites after initiation of ART during acute HIV.

## Subjects and Methods

### Patient Selection

This study investigated the first 40 enrollees of the RV 254/SEARCH 010 protocol, an ongoing study characterizing acute HIV infection in Bangkok, Thailand (clinicalTrials.gov # NCT00796146 and NCT00796263). All cases were enrolled during Fiebig stages I-IV, the earliest stage of HIV infection before appreciable antibody response can be detected and typically less than one month after exposure. Details are provided elsewhere [3], [17]. Briefly, all cases were identified from two facilities in Bangkok, Thailand and were age 18 years or older. Although exclusion criteria included major medical or psychiatric disorders that could potentially impact cognition, there were no individuals meeting these exclusion criteria. Among the first 40 enrollees, 8 were not evaluated in the MRS portion of the study due to conversion to Fiebig V before enrollment (n = 4) presence of metallic dental braces (n = 2), claustrophobia (n = 1) or no consent to MRS (n = 1). Additionally, one case was later excluded due to presumed neurosyphilis.

Immediately after enrollment and baseline MRI/MRS, 29/31 participants started ART composed of efavirenz (EFV), tenofovir (TNF), and emtricitabine (FTC) (standard ART). Eighteen of these subjects also started raltegravir (RAL) and maraviroc (MRC) (“mega-ART”). Efavirenz was discontinued in 9 cases due to intolerance (n = 7) or primary drug resistance (n = 2). Among these, only two cases were on standard ART, and the efavirenz was replaced with raltegravir. Seventeen subjects who completed all follow-up MRS (months 1, 3, and 6) were evaluated to investigate treatment response.

### Ethics Statement

The studies were approved by institutional review boards in Thailand (Chulalongkorn University Hospital, FWA00000943, IRB00001607) and in the U.S. (University of California, San Francisco, FWA00000068; and the Walter Reed Army Institute of Research, Rockville, MD, FWA00000015). All participants signed written consent.

### Chronic HIV-infected and HIV-negative Control Groups

Twenty-six ART-naïve chronically infected HIV+Thai subjects (participating in one of two studies: clinicalTrials.gov # NCT00782808 or NCT00777426) and 10 HIV-negative Thai controls (screened by a trained clinician to be ‘healthy’) were analyzed for comparison. All controls and chronically infected subjects were deemed to have normal cognition after neuropsychological testing and multi-disciplinary consensus conferences, which included a neurologist and a neuropsychologist, met the same exclusion criteria as the acute subjects, and were free of opportunistic infection. All subjects underwent one-time (baseline) MRS using the same protocol on the same scanner with no interim software or hardware upgrades and captured by the same technician (MP).

### Clinical Parameters

Lymphocyte subsets were measured at a local clinical laboratory and plasma HIV RNA levels were measured in fresh specimens by Roche Amplicor HIV-1 Monitor Test v1.5 with dilution for HIV RNA>750,000 copies/ml.

### Imaging Acquisition and Quality Control

**Figure 1:**
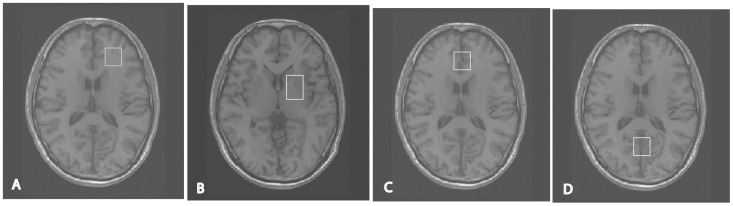
MRS voxel locations. A) left frontal white matter; B) basal ganglia; C) midline frontal gray matter; D) occipital gray matter.

Subjects underwent an axial 3D T1-weighted spoiled gradient echo image (3D SPGR, TE = 7 ms, TR = 11.2 ms, flip angle = 25^o^, with 1 mm voxel resolution) MRI scan on a GE Signa HDx 1.5T clinical scanner (GE Healthcare, Milwaukee, WI, software version 12-M4) using an 8-channel head coil for data reception and a standard body coil for transmission. Single voxel MRS was acquired using a double spin echo data acquisition (PROBE-P) with TE = 35 ms, TR = 1.5s at four voxel locations: left frontal white matter (FWM, 8cc), midline frontal grey matter (FGM, 8cc), occipital grey matter (OGM, 8cc) and basal ganglia (BG, 12cc, **Figure 1**). A total of 16 unsuppressed water free induction decays (FIDs) and 128 water suppressed FIDs were acquired for all voxels, with 192 water suppressed FIDs acquired at BG. To ensure scanner stability, short echo-time (TE = 35 ms) single voxel MRS measurements using a standard spectroscopy phantom (GE Healthcare) were performed at the conclusion of each subject examination [18].

### MRS Processing and Metabolites Quantification

Data were transferred by secured protocol to the central processing location at Huntington Medical Research Institutes in Pasadena, CA where two authors (NS and WR) processed all data using the time domain linear combination fitting software, LCModel (version 6.2, http://s-provencher.com/pages/lcmodel.shtml). Briefly, time domain MRS data from each coil of the 8-channel phased array head coil were combined using unsuppressed water FIDs from each coil as scaling factor [19]. The FIDs were processed without spectral line broadening for fitting. Fittings were performed between 4.0-0.5 ppm, using a reference basis set acquired using the same data acquisition parameters as *in vivo*. The reference solutions were 50 mM NAA, 50 mM CHO, 100 mM GLU, 100 mM GLN, 100 mM CR, and 150 mM MI. All reference solutions were adjusted to pH 7.2 with 0.1 M NaOH. Metabolite quantification for NAA, CR, tCHO, MI, and GLX were included only if the signal to noise ratio was>4 and their percent standard deviations were<20% [20].

### Statistical Analysis

Baseline characteristics between groups were compared using analysis of variance (ANOVA) and Tukey procedure for pairwise comparisons. We analyzed the main effect of acute and chronic HIV on metabolites using general linear model multivariate analyses of variance (MANOVA). We completed multivariate analyses using Pillai’s Trace tests for each voxel including the three groups as predictors and the four metabolites of interest (NAA, MI, CHO, GLX) as responses. For voxels where we rejected the joint null hypothesis of no effect of acute, chronic and control groups on the responses at p = 0.05, we then examined the individual metabolite levels within those models. Voxels for which we did not reject the null hypothesis were not investigated further. For voxels for which the null hypotheses were rejected, further analyses compared the metabolites among the different groups using ANOVA. In all models, CR and age were included as covariates. Although mean educational attainment and gender differed among groups, they were not included in the primary models since our main analyses compared acute cases to controls and since age and CR were more important to covary than was education and gender, each was felt less likely to appreciably impact MRS. Secondary analyses included education.

For the acute HIV+group, we used Spearman’s correlation to evaluate the relationship between MRS data and clinical variables. We evaluated MRS changes over time using mixed models with a complete case analysis approach. The 6-month metabolite ratios were compared to the cross-sectional HIV-negative control ratios to assess for normalization of values associated with treatment.

## Results

### Clinical Composition

The 31 acute HIV+subjects were at Fiebig stages I (10); II (3); III (15); and IV (3) at the time of MRS with a median (range) estimated duration since exposure of 14 (1–32) days (**Table 1**). Individuals tended to be young men who have sex with men (MSM) and no subjects reported injection drug use. In cases that provided information, 14 subjects denied illicit drugs for at least four months prior to enrollment, two were active users of methamphetamine, and nine were non-illicit drug users. The median (range) CD4^+^lymphocyte count and plasma HIV RNA level were lower for the chronic HIV group (p<0.0001 and p = 0.014, respectively). The HIV subtypes were identified to be CRF01_AE (n = 25) and B (n = 1) with 5 cases that could not be typed.

**Table 1:** Demographic characteristics of the investigated groups.

Results	Acute (n = 31)	Chronic (n = 26)	Control (n = 10)	P-value
Gender	84% male	50% male	60% male	0.022
Age, yrs, mean (range)	30 (19–46)	34 (25–48)	36 (27–45)	0.0340
Education, yrs, mean (range)	16 (6–25)	11 (3–19)	16 (6–25)	0.0002
Risk category ^1^				
-Homosexual	6	13	**–**	
-Heterosexual	–	1	**–**	
-Bisexual	22	10	**–**	
-Transfusion	–	2	**–**	
-Unknown	3	–	**–**	
Infection duration ^2^, days, median (range)	14.0 (1–32)	**–**	**–**	
CD4 count, cells/mm3, median (range)	428 (132–1127)	215 (19–553)	**–**	<.0001
Log10 plasma HIV RNA, median (range)	5.51 (2.78–7.41)	4.75 (3.22–5.88)	**–**	0.0142
Log10 CSF HIV RNA, median (range)^3^	3.39 (1.70–5.12)	**–**	**–**	

1. Risk factors by self-report;

2. Self-reported days since estimated exposure at enrollment [when a range of dates was provided (multiple potential exposures) the mean was used]. For age, chronic cases and controls do not differ, but each differ from mean of acute cases; for education, acute cases and controls do not differ, but each differ from chronic cases;

3. Data available on 24/31 cases.

### Scanner Quality Assurance

A total of 133 MRI/MRS examinations were performed on 67 subjects over a period of 32 months (from December 2008 to May 2011). We observed low percent coefficient of variation of phantom metabolite concentrations (Baseline: NAA/CR = 2.56%, CHO/CR = 2.62%, MI/CR = 3.33%, NAA/MI = 3.56%; 6 months: NAA/CR = 2.60%, CHO/CR = 2.65%, MI/CR = 3.40%, NAA/MI = 3.70%) reflecting hardware stability and an ability to reliably detect changes in metabolite ratios of 2–4%.

### Baseline Brain Metabolites

Our statistical approach identified voxels at BG (*p* = 0.002) and OGM (*p* = 0.037) for further investigation (**Table 2**). In BG, we identified increased tCHO/CR in acute compared to control groups (0.249 vs. 0.227, *p* = 0.001) and compared to chronic groups (0.233, *p* = 0.002). NAA/CR did not differ compared to controls although it was higher than that measured in the chronic group (1.134 vs. 1.000, *p* = 0.005). In OGM, we similarly noted elevated tCHO/CR in acute compared to control groups (0.176 vs. 0.153, *p* = 0.001). We also identified higher GLX/CR in the acute compared to chronic groups (1.980 vs. 1.763, *p* = 0.004), but no significant differences between GLX/CR in control subjects and either HIV group. We did not include education in our primary models due to the small sample size; however, repeated analyses that included education did not appreciably alter these findings. Voxels at FWM and FGM were not evaluated as they did not meet scrutiny of our statistical approach. Secondary explorations in these voxels did not suggest missed findings.

**Table 2:** Metabolites at BG and OGM, adjusting for Cr and age.

	All (N = 67)		Acute (n = 31)		Chronic (n = 26)		Control (n = 10)		P-value^1^	P-value^ 2^	P-value^3^
	Mean	SD	Mean	SD	Mean	SD	Mean	SD			
**Basal Ganglia**											
MI	0.574	0.12	0.580	0.13	0.560	0.12	0.589	0.09	0.449	0.898	0.689
NAA	1.073	0.15	1.134	0.14	1.000	0.14	1.077	0.13	0.0050.0051	0.552	0.014
tCHO	0.239	0.02	0.249	0.02	0.233	0.02	0.227	0.01	0.002	0.001	0.001
GLX	1.880	0.29	1.963	0.34	1.807	0.23	1.812	0.22	0.062	0.290	0.146
Multivariate Statistics											
Pillai’s Trace									<0.001	0.016	0.002
**Occipital Gray Matter**											
MI	0.766	0.08	0.765	0.08	0.773	0.09	0.751	0.10	0.707	0.353	0.554
NAA	1.417	0.12	1.416	0.13	1.415	0.12	1.428	0.12	0.958	0.123	0.323
tCHO	0.170	0.02	0.176	0.02	0.168	0.02	0.153	0.02	0.162	0.001	0.015
GLX	1.875	0.28	1.980	0.21	1.763	0.34	1.841	0.23	0.004	0.052	<0.001
Multivariate Statistics											
Pillai’s Trace									0.069	0.041	0.035

1, p-value for comparison between acute and chronic HIV;

2. p-value for comparison between acute and control;

3. p-value for comparison among acute, chronic, and control groups.

Multivariate models within the acute group identified a relationship between CSF HIV RNA and metabolites in FWM only. Examining individual metabolites at this voxel, we identified a direct association between log_10_ CSF HIV RNA and GLX/CR (β = 0.193, p = 0.002). We did not identify associations with estimated duration of infection. Patients with Fiebig III/IV had significantly increased BG NAA/CR after adjusting for age, as compared to patients with Fiebig I/II (β = 0.056, p = 0.018). No other correlations were identified in relation to Fiebig stage.

### Metabolite Changes with ART

**Figure 2:**
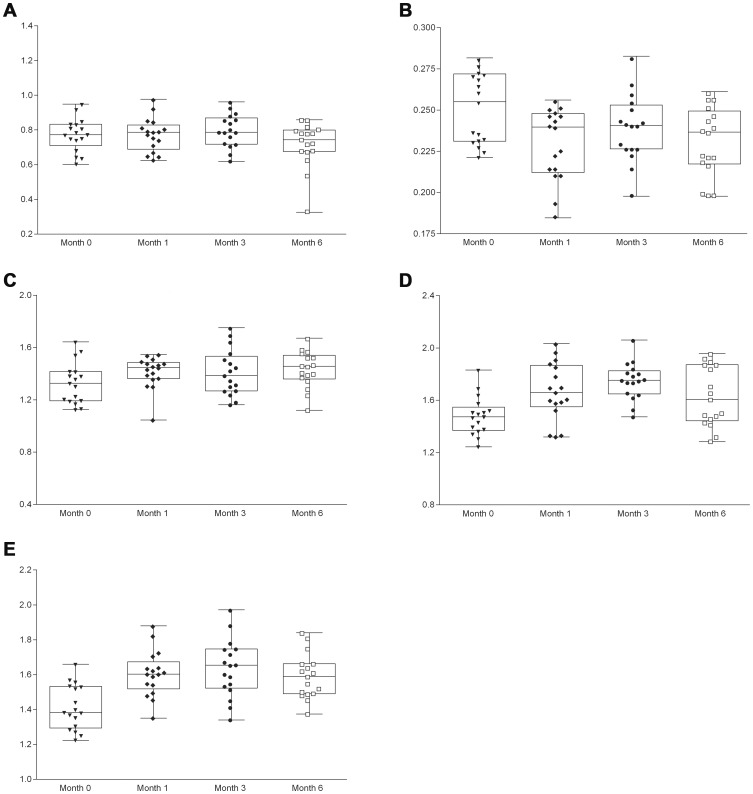
Metabolites demonstrating change associated with antiretroviral treatment. Metabolites demonstrating change associated with antiretroviral treatment included those demonstrating decreased inflammation [Panels A (MI/Cr in Occipital Grey Matter) and B (CHO/Cr in Basal Ganglia)] and those demonstrating increased neuronal integrity [Panels C (NAA/Cr in Frontal Grey Matter), D (NAA/Cr in Frontal White Matter), and E (NAA/Cr in Occipital Grey Matter)].

In longitudinal mixed models, we identified two categories of change following treatment with ART. Decreased inflammatory metabolites were noted with tCHO/CR at BG (*β* = −0.002/month, *p* = 0.022) and MI/CR at OGM (*β* = −0.009, *p* = 0.029). Increased NAA/CR was noted in FGM ((*β* = 0.013/month, *p* = 0.038), FWM (*β* = 0.018/month, *p* = 0.032) and OGM (*β* = 0.021/month, *p*<0.001) longitudinally. Most of these changes were temporally related to initiation of treatment with large changes at month 1 and lesser changes thereafter (**Figure 2**). We also noted two distinct groups of subjects relative to CHO/CR in BG. Using Pearson correlations we investigated the relationship between baseline CHO/CR and that at 1, 3, and 6 months identifying the following correlation coefficients and p-values: r = 0.544, p = 0.024; r = 0.761, p<0.001; and r = 0.449, p = 0.070, respectively, suggesting that individuals with higher values at baseline tended to also have higher values at follow-up. The MRS signature at six months following ART initiation did not differ from that of controls, adjusting for age and comparing all metabolites at each voxel.

## Discussion

Findings from this study provide indirect evidence for inflammatory changes within brain parenchyma during the earliest stages (median 14 days after HIV transmission) of HIV infection. Our findings extend existing knowledge of brain involvement through CSF analyses by demonstrating abnormalities within brain tissue itself. In our work, the MRS inflammatory signature normalized after ART that included medications with high (EFV, MVC, RAL, FTC) CNS penetration-effectiveness, based on cerebrospinal fluid studies. We noted no detectable differences seen in comparison to control subjects. The metabolite primarily altered was CHO, thought to reflect reactive cell turnover as it is closely correlated to membrane lipids [21]. Our findings suggest rapid lipid membrane turnover indicative of cellular inflammation in basal ganglia and posterior gray matter without appreciable change in metabolites referable to gliosis or neuronal injury. Although it has been suggested that alterations in CHO/CR may be reflective of change in brain volumes, it is not likely to reflect atrophy in our work given the acute stage of disease in otherwise healthy subjects [22]. The novelty of this finding relates to the very early evaluation in humans; however, a limitation relates to other potential factors that may influence the metabolic markers, particularly methamphetamine use in some cases. Exclusion of cases known to have active methamphetamine use at entry (n = 2) did not appreciably alter our findings; although significance crossed 0.05 for Pillai’s trace on model 2 (to 0.0535). Based on published work, gender should not have influenced metabolite levels in the voxels studied despite differences noted in acute cases (84% male) compared to controls (50% male, p = 0.006). [23] We also note comparison to healthy Thai controls and do not have cases of non-virally induced neurological damage, limiting our ability to focus attribution on viral mechanisms.

Unexpectedly, we noted a rise in NAA/CR in several brain regions associated with treatment. Despite this finding, neither the baseline nor the six-month NAA/CR ratios differed in the acute compared to control groups. Recognizing the risk that the trend could be an artifact of using CR as a standardizing metabolite, we subsequently evaluated NAA concentrations (uncorrected for T1 and T2 relaxation times) at these voxels, confirming a rise of NAA at BG but not at other locations. Since neither NAA nor NAA/CR differed from that of control subjects at any time point, the clinical significance of this finding may be less important and speculation regarding etiology premature. Our study design was limited by having control subjects imaged only once (baseline) with our comparison to acute cases who were imaged longitudinally.

We did not identify a decrease in markers of neuronal injury in early HIV. Our study subjects were imaged substantially earlier than those included in a prior report demonstrating reduced NAA/CR ratios in early infection in which subjects were enrolled up to 90 days after Western blot evidence of infection [5]. Furthermore, our subjects were neurologically asymptomatic. Simian models also have identified early neuronal injury by MRS; but, these models employ neurovirulent viral strains and immune depleted macaques, thus, may be less reflective of natural infection in humans where mucosal infection results in viral selection (transmitted viruses) and the inoculation may be smaller [24]. HIV subtypes in U.S. studies are likely to be type B, whereas most individuals in our Thai cohort are infected with a recombinant form of A and E viruses (CRF AE_01), a factor that could influence neurovirulence.

The differences observed in NAA/CR and GLX/CR between acute and chronic groups is likely related to smaller bidirectional alterations in both acute and chronic groups relative to the control group, although, this is speculative since no statistically significant differences to controls was identified in either group. In contrast, a reduction of glutamate has been observed in cognitively normal chronic HIV+subjects and in other studies of early HIV, but with similar caveats that the timing was later than that accomplished in our study, and effects of variable ART use cannot be excluded [25], [26]. Overall, these data suggest that NAA is not substantially altered in very early HIV infection, providing theoretical evidence for a potential window of opportunity where intervention may preserve brain function, recognizing that identification of infection this early in the clinical setting is challenging. Since all cases were treated immediately upon detection, we cannot inform the duration of this window.

Similar to our longitudinal CHO data, a small decrease in MI/CR was noted in OGM after treatment. Here, however, neither baseline nor follow-up values differed statistically from that of our control group. Elevated MI has been observed in cognitively impaired HIV+subjects during the chronic stage of infection [21]. Consistent with other reports investigating early infection, we did not identify significant change in MI in acute HIV [5]. This likely reflects that the processes in the CNS during acute HIV infection do not include substantial gliosis [27].

Our observations support the hypothesis that immune cellular infiltration and turnover characterizes the brain during the earliest stage of HIV infection, providing an early conduit for brain parenchymal viral seeding [28]. Added to knowledge that HIV RNA is detected in CSF within the first week after infection, these data are indirect evidence that the CNS viral reservoir may be established within weeks of infection [3]. Our data inform emerging intervention strategies designed to cure HIV that are dependent on purging latent reservoirs. The broad distribution of CHO/CR noted at baseline may suggest selective vulnerability. This would have been more suggestive if the longitudinal values were mixed; however, we saw a tendency for the individuals with higher ratios at baseline to have higher ratios post-ART. Thus, the distribution may represent background host variability.

In this work, the duration of HIV and Fiebig stage did not appreciably predict levels of MRS metabolites. In FWM, CSF HIV RNA level correlated to higher GLX/CR, but we did not identify associations for any other voxel/metabolite pair. Together, these findings suggest that the inflammation noted during acute HIV is likely not dependent on the level of systemic antibody response, and that CSF HIV RNA may correlate with release of neurotoxins in the brain but otherwise is independent of brain inflammation in this early period.

We had anticipated that the largest metabolic changes would be seen in BG and FWM based on the known higher frequency of detection of HIV antigen in these regions at autopsy in chronic HIV without treatment [29]. While we found abnormalities in BG and OGM, none were noted in FWM. This suggests that BG and OGM have the highest degree of vulnerability for cellular infiltration during acute HIV with BG but not OGM remaining a site of high viral burden in chronic HIV. A recent report noted grey matter atrophy with expansion of the third ventricle at one year after infection [30]. Visual inspection of high-resolution T1-weighted and T2-weighted structural images did not identify abnormalities in our subjects.

In conclusion, this study identified indirect evidence for brain parenchymal cellular infiltration in the absence of neuronal dysfunction or gliosis during the first month following HIV infection. We further found that this process is not present following 6 months of ART initiated immediately after diagnosis. Our findings highlight a potential pathway for early seeding of HIV and initial steps toward establishment of latent viral reservoirs in CNS, and suggest that early ART may be neuroprotective as a means to prevent neuronal injury and ameliorate brain inflammation.
